# Revisiting the Middle Molecule Hypothesis of Uremic Toxicity: A Systematic Review of Beta 2 Microglobulin Population Kinetics and Large Scale Modeling of Hemodialysis Trials *In Silico*

**DOI:** 10.1371/journal.pone.0153157

**Published:** 2016-04-07

**Authors:** Maria Eleni Roumelioti, Thomas Nolin, Mark L. Unruh, Christos Argyropoulos

**Affiliations:** 1 Division of Nephrology, Department of Internal Medicine, University of New Mexico Health Sciences Center, School of Medicine, Albuquerque, NM, United States of America; 2 Department of Pharmacy and Therapeutics, and Renal-Electrolyte Division, Department of Medicine, University of Pittsburgh Schools of Pharmacy and Medicine, Pittsburgh, PA, United States of America; San Raffaele Hospital, ITALY

## Abstract

**Background:**

Beta-2 Microglobulin (β2M) is a prototypical “middle molecule” uremic toxin that has been associated with a higher risk of death in hemodialysis patients. A quantitative description of the relative importance of factors determining β2M concentrations among patients with impaired kidney function is currently lacking.

**Methods:**

Herein we undertook a systematic review of existing studies reporting patient level data concerning generation, elimination and distribution of β_2_M in order to develop a population model of β_2_M kinetics. We used this model and previously determined relationships between predialysis β_2_M concentration and survival, to simulate the population distribution of predialysis β_2_M and the associated relative risk (RR) of death in patients receiving conventional thrice-weekly hemodialysis with low flux (LF) and high flux (HF) dialyzers, short (SD) and long daily (LD) HF hemodialysis sessions and on-line hemodiafiltration at different levels of residual renal function (RRF).

**Results:**

We identified 9 studies of 106 individuals and 156 evaluations of or more compartmental kinetic parameters of β_2_M. These studies used a variety of experimental methods to determine β_2_M kinetics ranging from isotopic dilution to profiling of intra/inter dialytic concentration changes. Most of the patients (74/106) were on dialysis with minimal RRF, thus facilitating the estimation of non-renal elimination kinetics of β_2_M. In large scale (N = 10000) simulations of individuals drawn from the population of β_2_M kinetic parameters, we found that, higher dialytic removal materially affects β_2_M exposures only when RRF (renal clearance of β_2_M) was below 2 ml/min. In patients initiating conventional HF hemodialysis, total loss of RRF was predicted to be associated with a RR of death of more than 20%. Hemodiafiltration and daily dialysis may decrease the high risk of death of anuric patients by 10% relative to conventional, thrice weekly HF dialysis. Only daily long sessions of hemodialysis consistently reduced mortality risk between 7–19% across the range of β_2_M generation rate.

**Conclusions:**

Preservation of RRF should be considered one of the therapeutic goals of hemodialysis practice. Randomized controlled trials of novel dialysis modalities may require large sample sizes to detect an effect on clinical outcomes even if they enroll anuric patients. The developed population model for β_2_M may allow personalization of hemodialysis prescription and/or facilitate the design of such studies by identifying patients with higher β_2_M generation rate.

## Introduction

Beta 2 Microglobulin (β_2_M) is an 11.6 kDa protein expressed in the surface of every nucleated cell, where it non-covalently associates with the alpha-chain of the Major Histocompatibility Complex I (MHC-I)/Human Leukocyte Antigen I (HLA-I) to facilitate antigen presentation. [1,2] It has long been appreciated that glomerular filtration is the major pathway for the elimination of β_2_M. [3–7] Residual renal function (RRF), inflammation and malnutrition appear to affect β_2_M concentration in patients with chronic kidney disease (CKD) [8–11] and end-stage renal disease (ESRD) [12–17]

The main recognized manifestation of β_2_M accumulation in patients receiving long-term dialysis is *dialysis-related amyloidosis* [18–22], but more recently β_2_M has also been linked to higher mortality in hemodialysis (HD) patients [14,23,24], to aortic calcification and cardiovascular mortality in patients with non-dialysis dependent CKD [25]. Nevertheless, more efficient dialytic removal of β_2_M has not equivocally translated into improved outcomes in randomized controlled trials (RCT) of *High Flux* membranes [14,26] and on-line hemodiafiltration (HDF) [27–29] creating uncertainty regarding the clinical effectiveness of enhanced middle molecule removal. Understanding of these discrepant findings and their implication for the middle molecule toxin theory that has been the driving biological hypothesis for the majority of randomized trials to date, requires one to simultaneously consider the ability of the available dialysis modalities to remove β_2_M and the considerable intra-individual, biological, variability in the kinetics of β_2_M. However, a quantitative description of the relative importance of intra-individual factors determining β2M concentrations among patients with impaired kidney function is currently lacking.

This report aims to develop a population kinetic model incorporating the intra-individual variability in generation, distribution and extrarenal elimination of β_2_M, which is then used to describe the disposition of β_2_M under different HD regimes and levels of RRF. To develop this population kinetic model, we first undertook a *patient-level* review and synthesis of the literature of clinical studies (either observational or interventional) regarding these kinetic parameters of β_2_M in humans. We used these parameters to simulate β_2_M concentrations and relative survival in a *population of ESRD patients* with different levels of RRF, using previously reported dose response relationships between predialysis β_2_M concentration and survival [30]. In these simulations we contrasted the intervention protocols utilized in RCTs of HD patients vis-à-vis RRF as determinants of patient survival.

These large scale simulations allowed us to conduct *in-silico* randomized controlled trials of different dialysis modalities i.e. low flux (LF, negligible β_2_M clearance) and high flux (HF, higher β_2_M clearance) membranes in conventional thrice-weekly HD, HF dialysis in short and long daily sessions and HDF. Our simulations not only recapitulate the design and findings of previously reported trials in dialysis, but also generate hypotheses about novel targets of intervention in nephrology and trial designs for the validation of the middle molecule hypothesis.

## Materials and Methods

### Systematic review of studies of β_2_M kinetics

These were identified by searching MEDLINE with the following (text) string: “(beta 2 microglobulin) AND (kinetic OR kinetics OR model OR models) AND (mathematical OR compartmental OR compartment OR simulation) AND (volume OR generation OR clearance OR dialysis OR renal OR dialytic OR production)” supplemented by manual inspection of the bibliography of indentified papers in a previous narrative review. [31] We did not screen articles but proceeded to full text of all studies indentified through the Medline search, to exclude those that were review articles, *in-vitro* or animal investigations, simulation experiments, failure to employ a kinetic model, or reported aggregate rather than subject level data (exclusion criteria). We included papers if they had used a compartmental model to study β_2_M kinetics, reported patient-level data and were published in English prior to 2015. As this was not an outcomes systematic review, we did not register our systematic review prospectively. Two investigators (MR, and CA) jointly extracted the data (values of kinetic parameters about the generation, intra-compartmental distribution, volume of compartments, non-renal clearance of β_2_M) from each individual participant in each study using a piloted form. This form and the patient level data extracted from our evidence synthesis are given in S1 Table.

Since classical techniques for the assessment of bias e.g. funnel plots are not applicable when the studies synthesized lacks a discrete health outcome, we were not able to conduct a formal analysis of bias with these methods. However for each study we evaluated the number of parameters reported, those fixed and those unreported by the investigators as an indicator for bias. We considered studies that did not report (or fixed to a specific value) of at most one parameter as studies with minimal risk of bias. As the number of parameters with fixed (or unreported values) increases, the estimated values of the remaining parameters becomes more and more dependent on the specific assumptions of the investigators and thus the risk for bias increases. Further details are provided in the Prisma Checklist (S2 Text).

### Compartmental simulation modeling

B_2_M kinetic simulations were based on the variable volume model [32–34] with two compartments (S1 Text and S1 Fig) incorporating inter and intra-dialytic generation, residual renal clearance, non-renal (extrarenal) clearance and dialytic routes of elimination. We simulated the kinetic parameters of 10,000 patients from the scaled–for–weight distributions of the population mean and standard deviation estimated from the literature synthesis at different levels of RRF (0–10 ml/min) and under regimes of conventional, thrice-weekly HD with either LF or HF dialyzers, short and long-daily HF HD and on-line HDF. Dialysis-related parameters reflected the patterns observed in FHN [35,36], HEMO [37] and HDF trials (Dutch CONTRAST [27], Spanish ESHOL [28], Turkish OL-HDF [29]), with specific details in Supplementary Methods in S1 Text. The purpose of these large scale simulations of patients with their unique set of generation/distribution/extrarenal clearance parameters and dialysis settings (dialyzer specifications, treatment time, blood flow rate, ultrafiltration and infusion rate of replacement fluid) was to summarize the effects of different interventions on β_2_M exposure.

### Statistical Analysis

#### Estimation of population kinetic parameters

We analyzed studies that collected multiple measurements in the same individual with a bi-level mixed-effect model accounting for individual (first level) and study (second level) heterogeneity; all other studies were analyzed with a random-effects model with a single (study) random effects model. Parameters were log-transformed prior to mixed-effect modeling of the population mean and (log-) variance, which was estimated by the between individual (two level models) or within study (one level model) standard error. Results are reported as means (SE) for the mean and the logarithm of the standard deviation of each log-transformed kinetic parameter. Furthermore, we calculated the population distribution of the untransformed parameters by transforming out of the logarithm and using the properties of the lognormal distribution. The volume of the two distribution compartments of β_2_M were analyzed both as absolute as scaled (to body weight) numbers. No other analyses e.g. meta-regression were performed on these data.

#### Analyses of simulation modeling results

We assessed treatment related exposures to β_2_M under time-dependent and peak-dependent toxicity perspectives by computing weekly Time Averaged Concentrations (TAC) and mid-weekly pre-dialysis plasma (C_p_) concentrations respectively. The relationship between TAC and C_p_ was analyzed via linear regression for all combinations of dialysis interventions and RRF levels. Dialytic interventions were compared on the basis of both TAC and C_p_ with a paired t-test. We assumed the following relationship between the relative risk (RR) of death and quintiles of cumulative predialysis β_2_M concentration observed over seven years in HEMO [30]: 1.0 (β_2_M< 27.5mg/l, referent), 1.11 (β_2_M:27.5-35mg/l), 1.35 (β_2_M:35–42.5mg/l), and 1.50 (β_2_M>42.5mg/l), obtained by averaging the risk in the last two quintiles due to the plateauing of the risk-exposure curve reported by the original investigators). Intra-individual changes in cumulative predialysis β_2_M concentration (averaged over the last two weeks of each simulation) between any two regimes in our simulations were thus converted to differences in RR. The latter, averaged over all individuals yield counterfactually the *Average Causal Effect (ACE)*. [38] We employed the connection between the ACE, a statistical measure of cause and effect, and the treatment effect estimated by RCTs [39] to interpret findings of recent trials and suggest hypotheses for testing in future studies. All analyses were performed in *R* (v2.15.1–3.1.3).

## Results

### Studies

**Fig 1:**
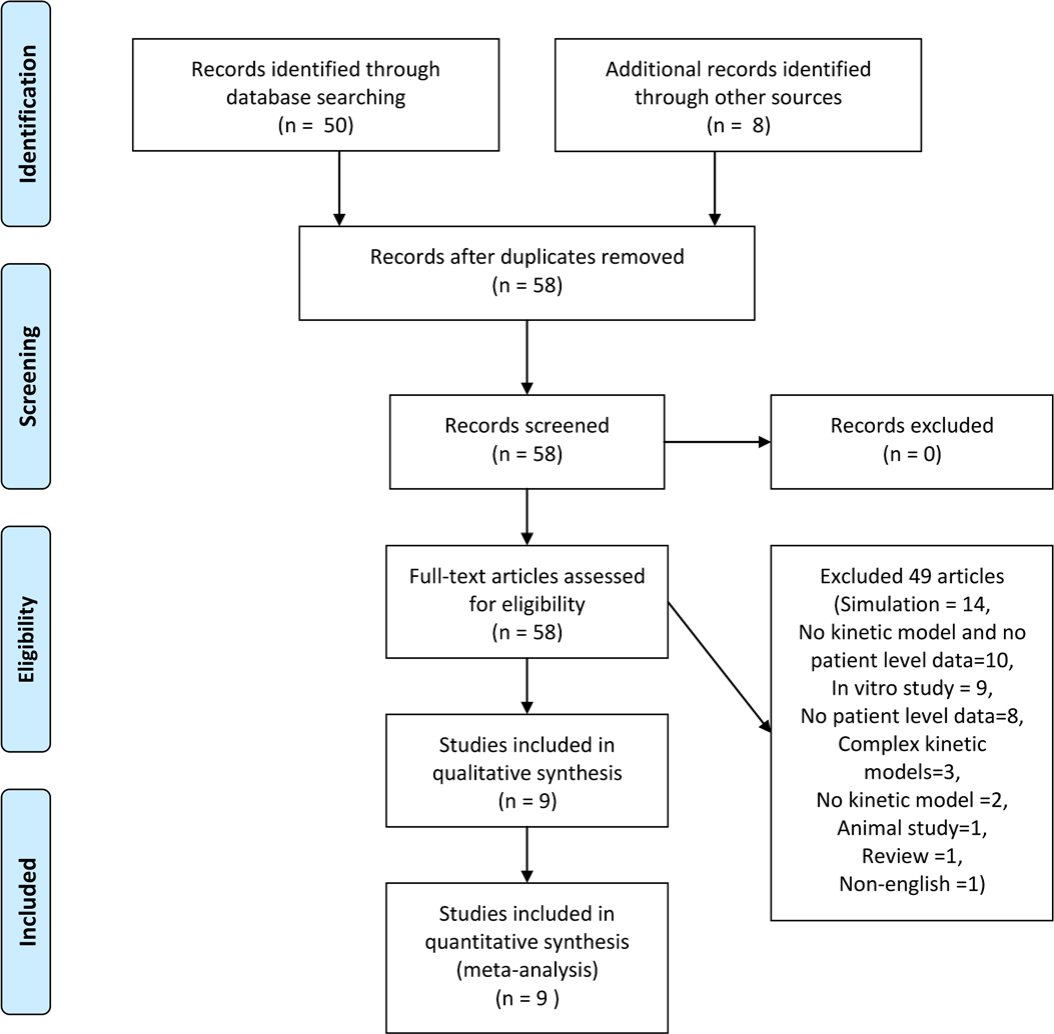
PRISM flow chart of the systematic review of human studies examining the compartmental kinetics of beta 2 microglobulin. A total of 58 studies were identified through a database (Medline) and other sources (examination of references of qualifying articles from the literature review, previous narrative review. A total of nine studies met all inclusion and none of the exclusion criteria.

A PUBMED search identified 57 papers (S2 Table). An additional eight papers were identified by manual searches of the bibliography of the identified papers and a previous narrative review[31]. Four papers out of the fifty identified through database searching fulfilled the criteria for inclusion and met no criteria for exclusion after full text review (see S2 Table for the references of these fifty papers and the inclusion/exclusion status). Three of the eight papers identified through other sources were excluded due to the use of highly compartmentalized, models that could not be reduced to the model utilized in this report [40,41],[42] yielding a total of nine separate studies (Fig 1). Four of the studies were at minimal risk of bias (one or none fixed/unreported parameters). Studies included in the systematic review, used experimental methods ranging from isotopic dilution to profiling of intra/inter dialytic concentration changes (Table 1) to determine β_2_M kinetics. In total, these studies reported 156 evaluations of one or more parameters of the compartmental model of β_2_M in 106 patients (Table 1). Most of the patients (74/106) were on dialysis with minimal RRF, facilitating thus the estimation of the non-renal (“extrarenal” [33]) clearance (*K*_*ER*_) of β_2_M. In subjects with normal kidney function, separate estimation of renal clearance (*K*_*R*_*)* and *K*_*ER*_ clearances is not possible, so that only a total body clearance (equal to *K*_*R*_*+K*_*ER*_) can be estimated.

**Table 1:** Published patient level data regarding β_2_ microglobulin kinetics.

Study	Number: Measurements /patients	Groups (N)	Kinetic Model	Parameters		
				Estimated & Reported	Fixed	Unreported
Karlson et al 1980[43]°	12 / 12	Control (6) MM (2) RA (1) CGN (2) RI (1)	2C	*G*, *K*_*IC*,_ *K*_*R*_*+K*_*ER*,_ *V*_*P*_, *BW*	None	*V*_*NP*_
Vincent et al 1980 [44]*	12 / 12	Control (2) HD (2) CKD (4) Transplant (4)	2C	*G*, *K*_*IC*,_ *K*_*R*_*+K*_*ER*,_ *V*_*P*_, *BW*	None	*V*_*NP*_
Maeda et al 1990[45] ^╪^	11 / 11	HDF (11)	1C	*G*, *BW*	*V*_*T*_	None
Floege et al 1991[46] ^¶^	16 / 16	Normal (5) LF-HD (6) HF-HD (5)	2C	*G*, *K*_*IC*,_ *K*_*ER*_, *BW*	*V*_*P*_	*V*_*NP*_
Odell et al 1991[47] ^┼^	8 / 5	LF-HD (4) HF-HD (4)	3C	*G*, *K*_*IC*,_ *K*_*ER*,_ *V*_*P*,_ *V*_*NP*_, *BW*	None	None
Vincent et al 1992[48] ^§^	22 / 22	Normal (5) LF-HD (5) HF-HD (5) HDF (4) CAPD (3)	3C	*G*,*K*_*IC*_, *K*_*ER*_,*V*_*P*,_ *V*_*NP*_, *BW*	None	*K*_*IC*,_ *V*_*NP*_
Xu et al 2001[49] ^+^	50 /10	LF-HD (10) HF-HD (40)	2C	*G*, *K*_*ER*_, *BW*	*V*_*P*_,*V*_*NP*,_ *V*_*T*_, *K*_*IC*_	None
Stiller et al 2002[32] ^×^	15 / 8	HF-HD (15)	2C	*G*, *K*_*IC*_, *V*_*P*_,*V*_*P*_: *V*_*NP*_, *BW*	*K*_*ER*_	*V*_*P*_^,^*V*_*NP*_
Ward et al 2006[33] ^¤^	10 / 10	HF-HD (10)	2C	*G*, *K*_*IC*_, *V*_*T*_	*V*_*P*_: *V*_*NP*_, *K*_*ER*_	*BW*

*Abbreviations*: MM (Multiple Myeloma), RA (Rheumatoid Arthritis), CGN (Chronic Glomerulonephritis), RI (Renal Insufficiency), LF-HD (Low Flux Hemodialysis), HF-HD (High Flux Hemodialysis), HD (Hemodialysis), HDF (Hemodiafiltration), Continuous Ambulatory Peritoneal Dialysis (CAPD) 1-3C: Model with 1, 2 or 3 Compartments. *Kinetic Parameters*: β_2_ Microglobulin Generation Rate (*G*), Intercompartmental Exchange Rate Constant (*K*_*IC*_), Extrarenal Clearance (*K*_*ER*_), Renal Clearance (*K*_*R*_), Perfusing/Plasma/Primary Distribution Volume (*V*_*P*_), Non-Perfusing/Tissue/Extravascular Distribution Volume (*V*_*NP*_), Total Distribution Volume (*V*_*T*_ = *V*_*P*_ + *V*_*NP*_), Body Weight (*BW*).

Notes

°All subjects had simultaneous creatinine measurements that were ≤ 1.2 mg/dl. *V*_*NP*_ was calculated from the forward and reverse intercompartmental transfer constants reported in the paper, assuming a sieving coefficient equal to one.

* Patients had simultaneous β_2_ microglobulin and inulin clearance determinations. Only one of the four transplant patients had a normal inulin clearance, but this was determined just before an acute rejection episode. The flux of the patients on dialysis was not specified in the manuscript. *V*_*NP*_ was calculated from the forward and reverse intercompartmental transfer constants reported in the paper, assuming a sieving coefficient equal to one.

╪ Volume of distribution fixed to a multiple of the plasma volume using anthropometric and previous kinetic data [44].

¶ Fixed to anthropometric estimate for blood volume for normal individuals adjusted for hematocrit.

┼ Patients were receiving low flux dialysis in the first study, but high flux dialysis on the second study. Three patients were assessed on both high and low flux dialyzers. *V*_*NP*_ was calculated by summing the reported volumes of the two non plasma compartments. The overall intercompartmental rate transfer constant was set equal to the average of the fastest and the sum of the rate constants to the two non-vascular compartments.

§ Calculated from the reported value of *Vp* and the relative size of plasma and tissue pools.

+ only a single value for the extrarenal clearance was reported; generation rate was assumed not to be influenced by the change in flux; *V*_*T*_ was set equal to 40% of the anthropometrically estimated plasma water and the ratio *V*_*P*_ */ V*_*NP*_ was assumed to be equal to 1:4; *K*_*IC*_ was set equal to 50 ml/min for all patients.

× *K*_*ER*_ set to 3.13 ml/min for all patients. *V*_*P*_ and *V*_*NP*_ calculated from the reported total distribution volume and the ratio of the two compartments.

¤ The authors assumed a constant ratio *V*_*P*_ */ V*_*NP*_ equal to 1:3 and *K*_*ER*_ of 3 ml/min for all patients.

Patient’s weight was not reported in the manuscript.

### Population β_2_M Estimates

Parameter estimates, and the resultant population distribution values (median, upper and lower 2.5% tail) are summarized in Table 2. In normal subjects kidney function is the major determinant of total body clearance as the estimated median *K*_*R*_*+K*_*ER*,_ was equal to 90.43 ml/min vs. 2.92 ml/min for *K*_*ER*_ in patients on dialysis.

**Table 2:** β2 microglobulin population kinetic parameters and quantile values.

			Mixed Model Parameter Estimates¶		Population Distribution Values^╪^		
Kinetic Parameter	Number (studies)	Number: measurements/patients	Mean(SE)	Logarithm of the Standard Deviation (SE)	Median	Q025	Q975
Generation Rate (mg/kg/day)	8[32,43–49]	146 / 96	1.1 (0.08)	-1.1 (0.1)	3.01	1.57	5.78
Intracompartmental Rate Transfer (ml/min)	6[32,33,43,44,46,47]	73 / 63	4.23 (0.21)	-1.26 (0.25)	68.54	39.37	119.3
Extrarenal Clearance (ml/min)	6[44–49]	59 / 56	1.07 (0.13)	-0.95 (0.12)	2.92	1.37	6.25
Total Body Clearance in Controls (ml/min)*	3[43,46,48]	19 / 19	4.5 (0.17)	-1.65 (0.18)	90.43	62.1	131.7
Total Volume of Distribution (L)	6[32,33,43,44,47,48]	79 / 69	2.4 (0.06)	-1.31 (0.08)	11.14	6.57	18.90
Total Volume of Distribution (% BW)	5[32,43,44,47,48]	69 / 59	-1.73 (0.09)	-1.44 (0.11)	17.73	11.13	28.24
Perfusing Compartment Volume (% BW)	5[32,43,44,47,48]	69 / 59	-3.06 (0.15)	-1.89 (0.18)	4.67	3.47	6.28
Ratio Of Non-Perfusing to Perfusing Compartment Volume	5[32,43,44,47,48]	69 / 59	1 (0.15)	-1.53 (0.29)	2.72	1.78	4.15

Notes

¶ Parameters obtained by a linear mixed model for the logarithm of each kinetic parameter. The model assumes that each kinetic parameter follows a log normal distribution (or equivalently that their logarithms are normally distributed). For each kinetic parameter the location and the logarithm of the variance of the corresponding log-normal distribution was estimated via a mixed effects model.

* Excluding the two controls from the study by Vincent, Pozet and Revillard [44] since the values for these 2 individuals were 5–6 times smaller than the simultaneously determined (via inulin clearance) GFR values.

╪ Q025: lower 2.5% quantile, Q975: upper 2.5% quantile.

### Hemodialysis Simulations

#### Residual renal clearance is a major determinant of β_2_M concentration in dialysis patients

**Fig 2:**
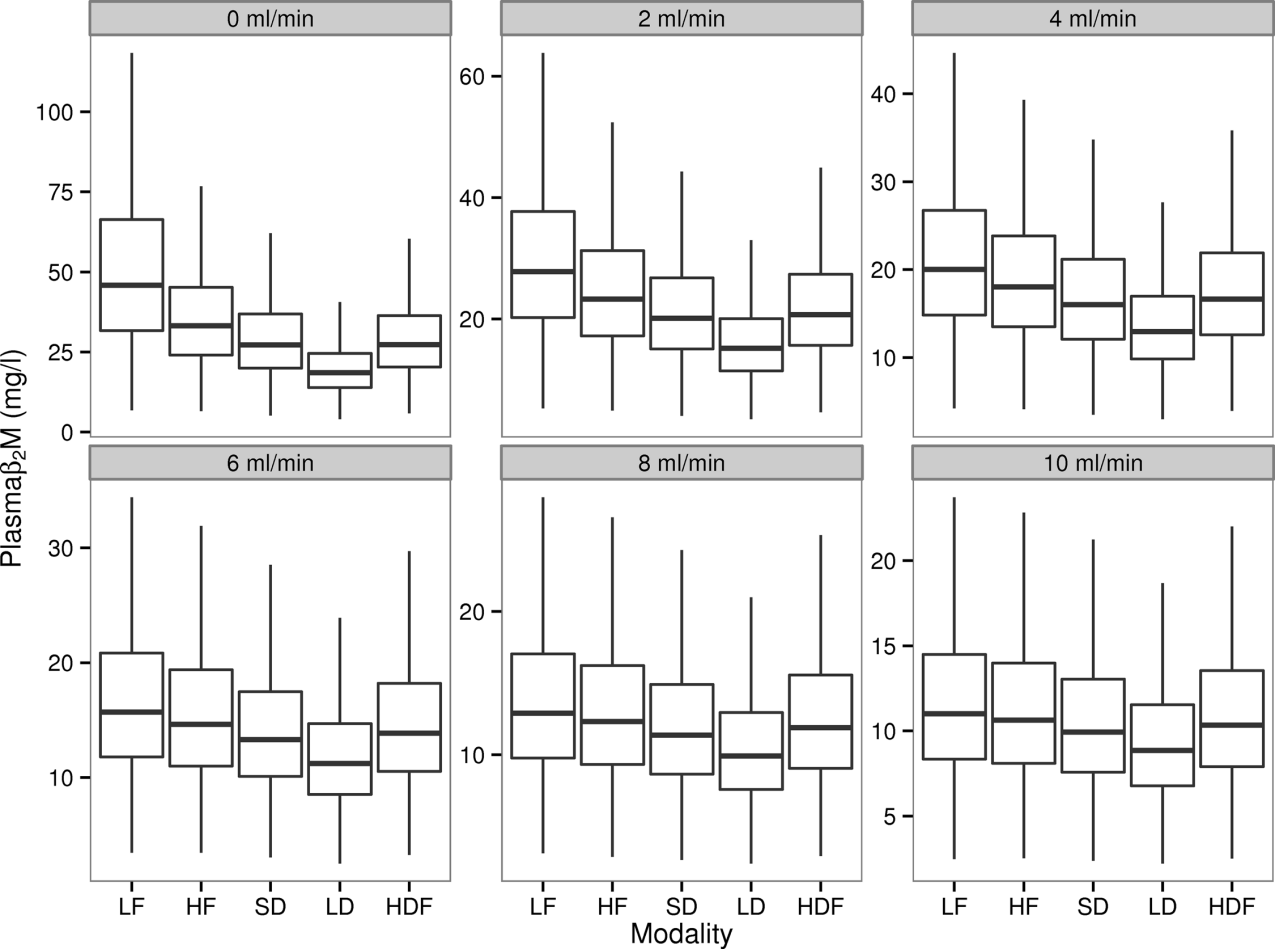
Simulated β_2_M concentrations at different levels of residual renal clearance and dialysis regimes. LF: Low Flux Dialysis, HF: High Flux Dialysis, SD: Short Daily Dialysis with High Flux dialyzers (6 times a week, ~ 2 ½ hrs per session), LD: Long Daily Dialysis with High Flux dialyzers (6 times a week, 6 ⅓ hrs per session), HDF = postdilution online hemodiafiltration.

The simulated predialysis β_2_M concentrations of different dialysis regimens at various levels of *K*_*R*_ are shown in Fig 2. The highest concentrations were seen in patients on LF HD irrespective of RRF due to minimal dialytic clearance. Conventional HD, short-daily or long-daily HF and HDF resulted in decreased β_2_M relative to LF HD. Time averaged concentrations (TAC) and predialysis concentrations were highly and positively correlated across all combinations of RRF and dialytic interventions (r^2^>0.99).

**Fig 3:**
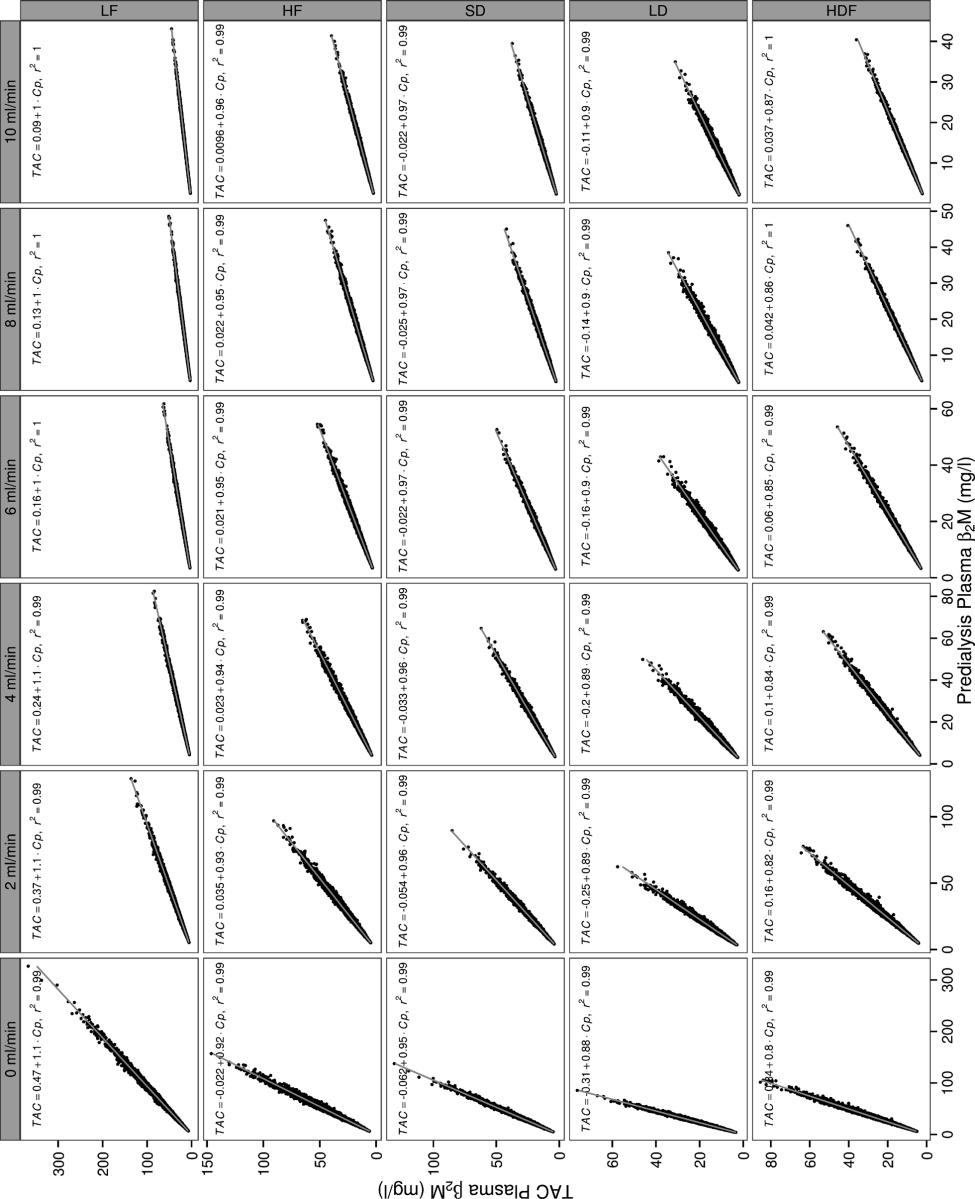
Relation between mid-week simulated predialysis and weekly time averaged β_2_M concentrations at different levels of residual renal clearance and dialysis regimes. Correlation coefficients (r^2^) and regression equations relating the two measures of exposure (gray line) are shown. LF: Low Flux Dialysis, HF: High Flux Dialysis, SD: Short Daily Dialysis with High Flux dialyzers (6 times a week, ~ 2 ½ hrs per session), LD: Long Daily Dialysis with High Flux dialyzers (6 times a week, 6 ⅓ hrs per session), HDF = post dilution online hemodiafiltration. Cp: predialysis plasma concentration, TAC: Time Averaged Concentration.

Regression analysis (Fig 3) also demonstrated a high numerical agreement between the two measures, with the slopes in linear regression being close to unity especially for higher levels of RRF. Nevertheless, this agreement differed for the different modalities; although the TAC was within 8% of the predialysis level for most modality, RRF combinations, it diverged more than 10% in long-daily or HDF at lower RRF levels.

Table 3 shows the pair-wise mean differences in β_2_M concentrations between different regimens at different levels of RRF. It is only in the absence of RRF (K_R_ = 0 ml/min) that higher dialytic removal of β_2_M in the form of short-daily (SD vs. HF, Table 3), or HDF (HDF vs HF, Table 3) had a substantial effect on β_2_M relative to conventional HF HD. Similarly, long daily dialysis consistently resulted in lower concentrations of β_2_M concentrations; the difference between the two modalities widened as renal function declined (LD vs. SD, Table 3). Finally, HDF resulted in predialysis β_2_M concentration that was very similar to the one achieved with short-daily dialysis at all levels of RRF (HDF vs SD Table 3).

**Table 3:** Mean pair wise difference in predialysis β2 microglobulin concentration (in mg/L) and associated 95% confidence interval as a function of residual renal clearance.

	HF vs LF		SD vs HF		LD vs SD		HDF vs HF		HDF vs SD	
K_R_ (ml/min)	Estimate	95%CI	Estimate	95% CI	Estimate	95% CI	Estimate	95% CI	Estimate	95% CI
0	-16.75	-16.45, -17.04	-7.51	-7.62, -7.41	-9.69	-9.80, -9.57	-7.47	-7.59, -4.47	0.04	-0.03, 0.12*
2	-5.00	-5.11, -4.90	-3.95	-4.00, -3.90	-5.42	-5.48, -5.36	-3.21	-3.26, -3.15	0.74	0.70, 0.79
4	-2.38	-2.42, -2.34	-2.45	-2.48, -2.42	-3.40	-3.44, -3.36	-1.64	-1.66, -1.61	0.81	0.78, 0.84
6	-1.25	-1.23, -1.27	-1.66	-1.68, -1.64	-2.28	-2.31, -2.26	-0.91	-0.93, -0.89	0.75	0.74, 0.77
8	-0.67	-0.69, -0.66	-1.20	-1.22, -1.19	-1.61	-1.63, -1.59	-0.55	-0.56, -0.54	0.65	0.64, 0.67
10	-0.40	-0.41, -0.40	-0.90	-0.92, -0.89	-1.18	-1.19, -1.16	-0.35	-0.36, -0.34	0.55	0.54, 0.57

*Notes*: Differences and 95% confidence intervals (95% CI) were computed by paired t-test; unless stated otherwise, p for all comparisons is <0.001

*p = 0.26

HF = High Flux thrice weekly dialysis, LF = Low Flux thrice weekly dialysis, SD = Short Daily dialysis, LD = Long Daily dialysis, HDF = post-dilution online hemodiafiltration

Comparisons based on TAC yielded congruent findings with respect to the relative efficiency of the different dialysis regimes and the role of RRF (Table 4). In these comparisons, the absolute difference in TAC (Table 4) between short-daily dialysis and HDF was much larger than the corresponding difference in C_p_ (shown in Table 3), suggesting that short-daily yields somewhat lower exposures than HDF compared to the expectations based on predialysis concentrations.

**Table 4:** Mean pair wise difference in the Time Averaged Concentration (TAC) of β2 microglobulin (in mg/L) and associated 95% confidence interval as a function of residual renal clearance.

	HF vs LF		SD vs HF		LD vs SD		HDF vs HF		HDF vs SD	
K_R_(ml/min)	Estimate	95%CI	Estimate	95% CI	Estimate	95% CI	Estimate	95% CI	Estimate	95% CI
0	-23.58	-23.95, -23.21	-6.20	-6.23, -6.11	-10.74	-10.86, -10.62	-10.19	-10.32, -10.06	-3.99	-4.07, -3.91
2	-8.96	-9.07, -8.86	-3.19	-3.23, -3.15	-6.49	-6.56, -6.42	-5.31	-5.37, -5.25	-2.12	-2.16, -2.08
4	-4.82	-4.89, -4.78	-1.96	-1.99, -1.94	-4.38	-4.43, -4.34	-3.28	-3.31, -3.24	-1.31	-1.33, -1.28
6	-3.03	-3.07, -3.00	-1.33	-1.35, -1.32	-3.17	-3.20, -3.14	-2.22	-2.25, -2.20	-0.89	-0.91, -0.87
8	-2.89	-2.11, 2.07	-0.97	-0.98, -0.96	-2.41	-2.43, -2.39	-1.61	-1.63, -1.60	-0.64	-0.66, -0.63
10	-1.53	-1.54, -1.51	-0.74	-0.75, -0.73	-1.89	-1.91, -1.87	-1.22	-1.24, -1.21	-0.49	-0.50, -0.48

*Notes*: Differences and 95% confidence intervals (95% CI) were computed by paired t-test; p for all comparisons is <0.001. HF = High Flux thrice weekly dialysis, LF = Low Flux thrice weekly dialysis, SD = Short Daily dialysis, LD = Long Daily dialysis, HDF = post-dilution online hemodiafiltration.

#### Residual renal function is more important than enhanced dialytic removal in determining β_2_M related outcomes in hemodialysis

**Fig 4:**
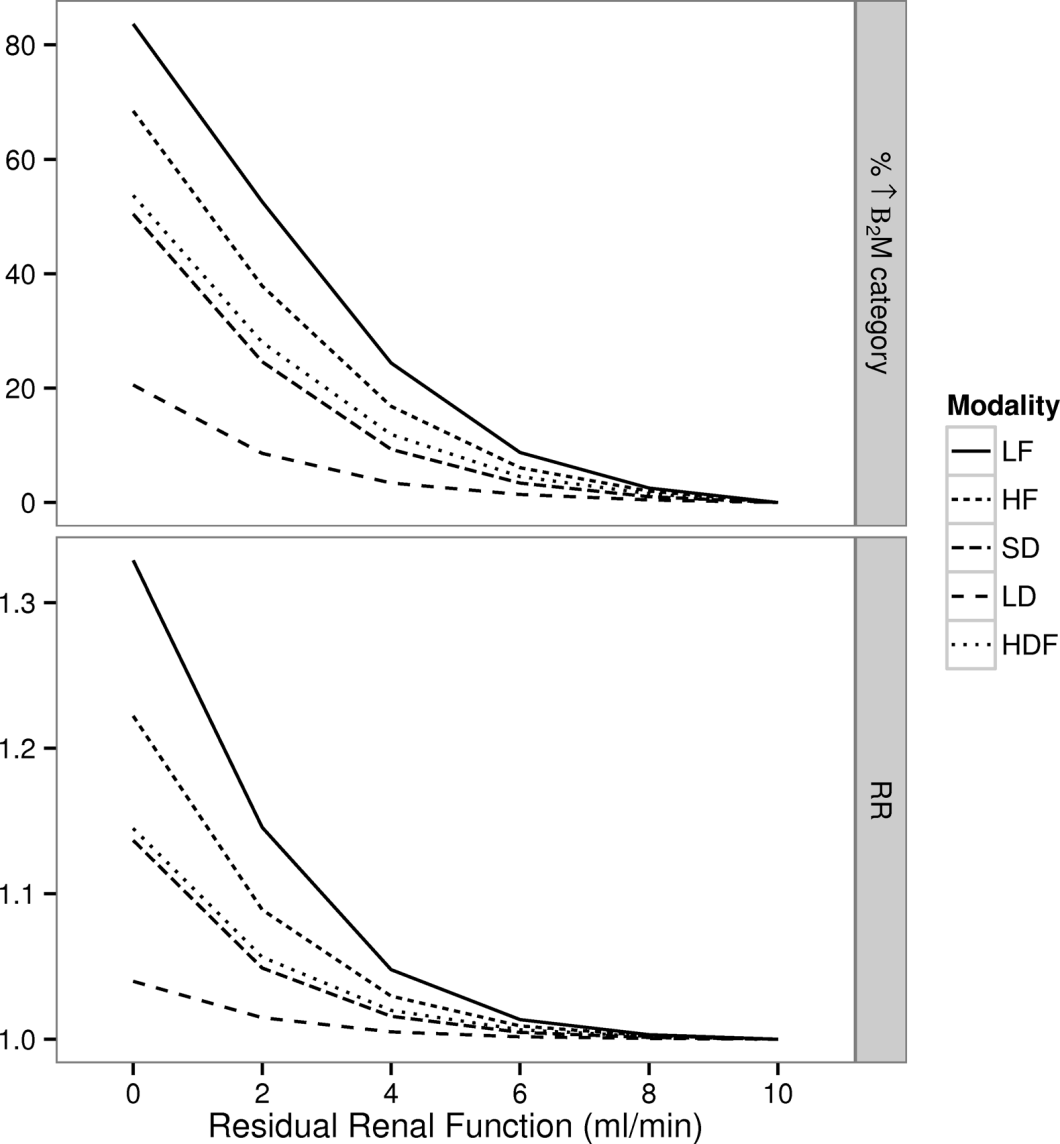
Simulated changes in β_2_M and predicted Relative Risk (RR) of death at different levels of renal function. For each dialysis regime we calculated: i) the percentage of patients undergoing a change in their cumulative predialysis β_2_M concentration (categorically classified as <27.5 mg/l, 27.5–35 mg/l, 35–42.5 mg/l and >42.5 mg/l) for the different levels of residual renal function (*K*_*R*_) relative to the baseline measurement when *K*_*R*_ = 10 ml/min ii) the associated prediction for the RR. Within each dialysis modality, the referent is the state with *K*_*R*_ = 10 ml/min

**Fig 5:**
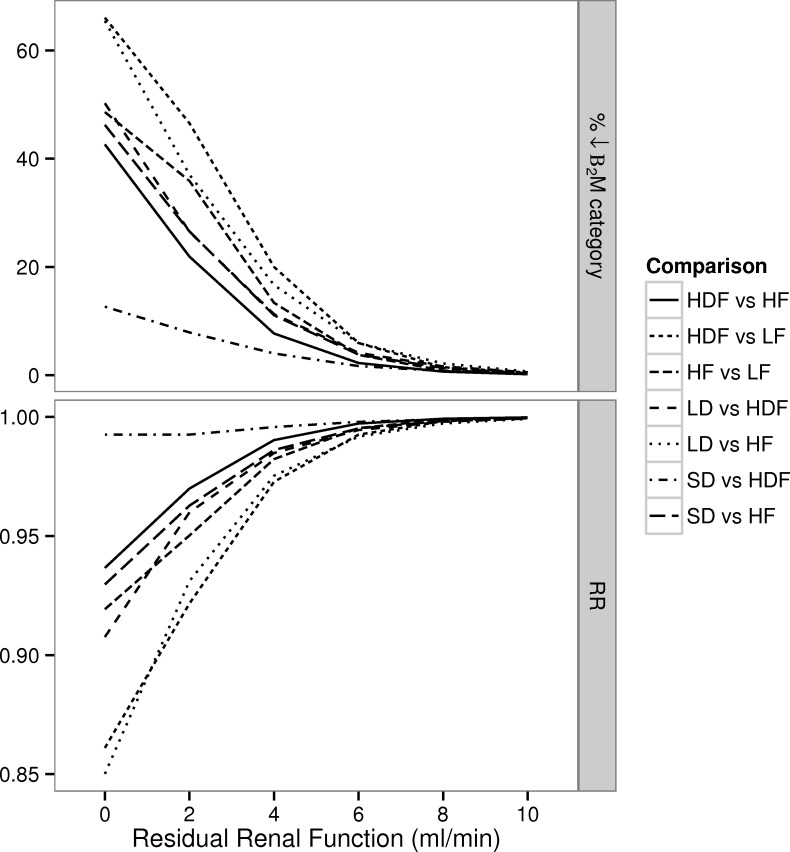
Simulated changes in β_2_M and predicted Relative Risk (RR) of death associated with enhanced dialytic removal. At each level of residual renal function (*K*_*R*_) we calculated: i) the percentage of patients with a change in the cumulative β_2_M concentration (categorically classified as <27.5 mg/l, 27.5–35 mg/l, 35–42.5 mg/l and >42.5 mg/l) between techniques of higher and lower dialytic removal of β_2_M ii) the associated prediction for the RR.

Reduction in residual *K*_*R*_ from 10 ml/min to nil resulted in an increase in the percentage of patients whose β_2_M concentration category (<27.5 mg/l, 27.5–35 mg/l, 35–42.5 mg/l and >42.5 mg/l) increased e.g. 20%-80% in patients receiving HDF and LF respectively. This was associated with an increase in the predicted RR (Fig 4), that was modified by the dialysis regime: 1.32 (LF), 1.21 (HF), 1.13 (SD), 1.03 (LD), and 1.13 (HDF). The magnitude of the RRs far exceeded the reductions in mortality that were predicted on the basis of enhanced dialytic removal of β_2_M (Fig 5), e.g. less than 8% for comparisons of HF vs. LF, short-daily or long-daily, and HDF vs. LF, HF or short-daily when *K*_*R*_ was 2 ml/min. In the absence of kidney function, the smallest RRs were seen in HDF and long-daily vs. LF and HF, and were in the order of 0.85. Under the remaining scenarios, dialysis with a regime that more efficiently removes β_2_M is predicted to be associated with single digit improvement in the RR.

**Fig 6:**
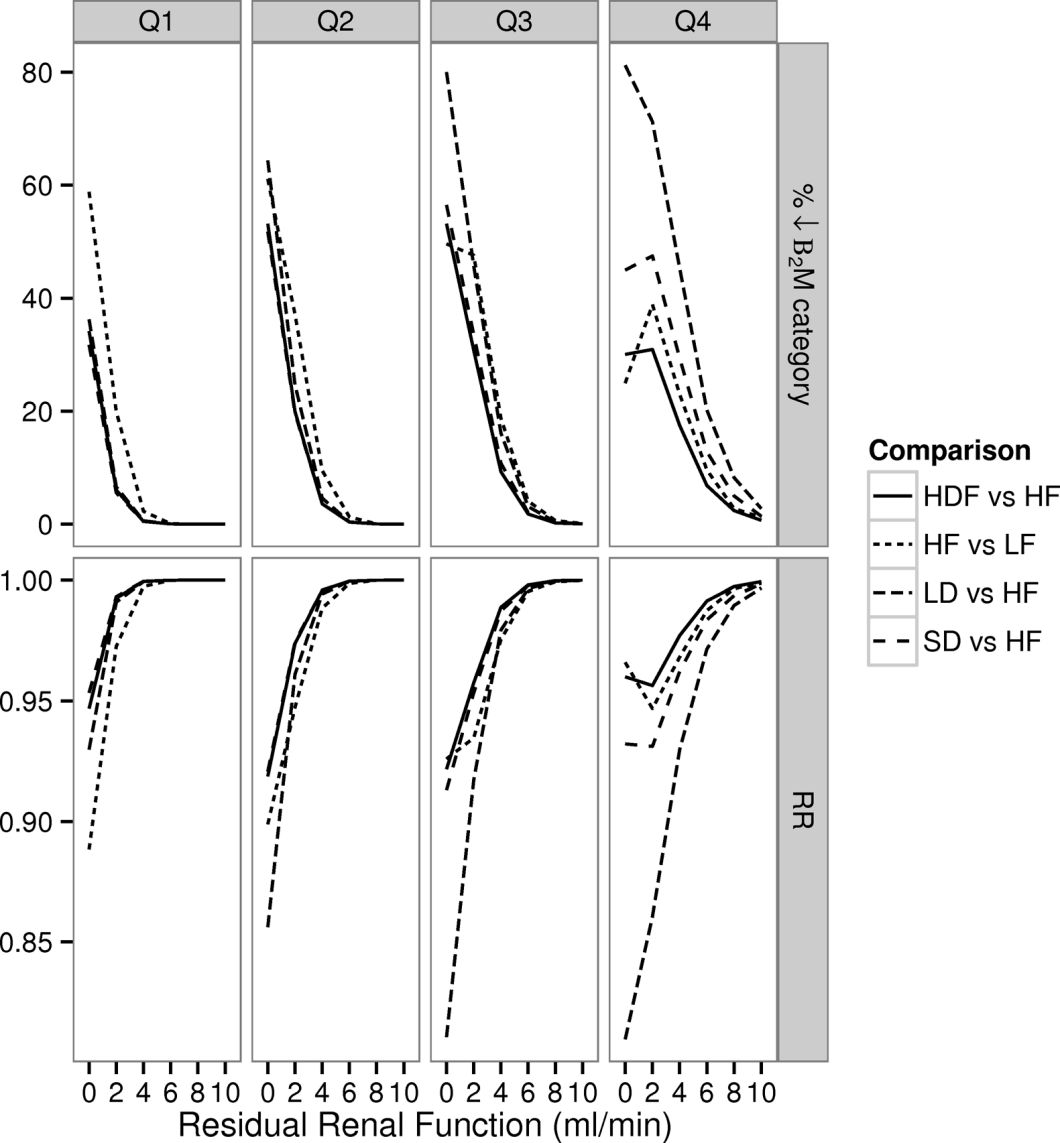
Simulated changes in β_2_M and predicted Relative Risk (RR) of death associated with enhanced dialytic removal at different quartiles (Q1-4) of generation rate. At each level of residual renal function (*K*_*R*_) and quartile of reuse we calculated: i) the percentage of patients with a change in the cumulative β_2_M concentration (categorically classified as <27.5 mg/l, 27.5–35 mg/l, 35–42.5 mg/l and >42.5 mg/l) between techniques of higher and lower dialytic removal of β_2_M ii) the associated prediction for the RR.

The estimated dialytic effects on β_2_M category and predicted survival were not uniform across subgroups defined on the basis of quartiles of increasing β_2_M generation rate. Relative to LF dialysis, adoption of HF membranes would be expected to reduce mortality by more than 10% in patients of the lower two (Q1-2) quartiles (Fig 6), but the effect is smaller and reaches a plateau for higher generation rates. A similar pattern was noted for HDF or short-daily vs. HF dialysis for the Q4 subgroup. Long-daily regimes are anticipated to improve outcomes more for patients at Q2-Q4 (15–19%) rather than those who generate β_2_M at the lowest generation rate (~7%). As anticipated dialysis effects were quantitatively more important for patients with higher generation at higher levels of RRF (i.e., the RR of 0.95 for short-daily vs. HF was observed for Q1 patients at a *K*_*R*_ of 0 ml/min vs. 10 ml/min for Q4 patients).

## Discussion

This report demonstrates estimated predialysis β_2_M concentrations averaging over the population variability in generation, distribution and extrarenal clearance of β_2_M under different levels of RRF. Since RRF is a major determinant of β_2_M in dialysis patients, increased dialytic removal becomes clinically important only when RRF has declined to below 2ml/min. Dialysis effects on survival outcomes related to β_2_M levels were moderate and heterogeneous in subgroups of patients of different generation rates at all levels of RRF and for a wide spectrum of dialysis regimens.

The population kinetic model reproduces experimental patterns *observed* in early studies of dialysis patients [13,30,31,50–52]. More specifically, we anticipated a large influence of RRF on plasma β2M concentration, particularly when the former declines below 2 ml/min. In retrospect, this prediction is not surprising but we derive this relation from first principles and in a quantitative fashion. This allows our numerical evaluations to generate testable hypotheses that can be verified or refuted by empirical data. In HEMO, predialysis β_2_M concentrations were higher in anuric patients [30] and declined curvilinearly for higher residual urea clearances (e.g. see Fig 3[30]), a pattern similar to the one described in this report. In the CONTRAST trial of HDF, the average β_2_M concentration in patients dialyzing with LF membranes and an eGFR of 2.0 ml/min/1.73 m^2^ *at baseline* was 30.7–32.3 mg/L, which is in close agreement with our estimate of 29.9 mg/L. [27] At a higher, semi-quantitative level, our analyses suggest that interventions that increase dialytic removal of β_2_M (i.e., use of HF membranes, addition of convective clearance in the form of HDF, increase in both frequency and duration of treatments) will be masked until RRF is substantially reduced in accordance with reports in HF dialysis [51] and on-line HDF [13,16].

Our findings provide a framework to reconcile reports suggesting that higher dialytic removal (HDF vs. HF) does not have a substantial impact on predialysis β2M concentration [28,29,53]. These discrepancies can be understood by highlighting the importance of both RRF and the (unmeasured) generation rate as determinants of the β_2_M response to dialysis. These HDF studies enrolled prevalent patients (median time on dialysis: 28–68 months) and with the exception of the study by Ward, [53] provided minimal data on RRF. Hence, in studies inferring the response of β_2_M to more efficient dialysis one should adjust for these parameters either directly by measuring RRF, or indirectly by using additional patient level covariates that potentially correlate with the generation rate of middle molecules.

Another novel feature of this report is the adoption of a counterfactual perspective in the population-level evaluation of different dialysis regimes. This perspective allowed us to predict relative changes in survival associated with changes in β_2_M (the prototypical middle molecule [54]) exposure. In ESRD more robust evidence that higher β_2_M concentrations are associated with worse survival comes from the HEMO study cohort, [23,30] in which β_2_M levels were assessed prospectively and repeatedly over time. The apparent dose response relationship in HEMO was observed in a cohort of patients with negligible RRF (only 14% of the 1704 patients had RRF >0.75 ml/min at study enrollment), and was detected with time-updated survival models in the presence of extensive multivariable adjustment including RRF. On the other hand, cross-sectional, observational studies utilizing single measurements of β_2_M yielded partially conflicting associations [24],[55]. More recently, associations have been reported between β_2_M and survival (all-cause and cardiovascular in NHANES [56] and ARIC [57]), cardiovascular events and calcification in CKD [25], early-onset atherosclerosis in ESRD [58], stroke [59], peripheral arterial disease [60–63], and mortality in patients undergoing coronary angiography. [64] Although observational, these associations have held against adjustments for known risk factors and the prevailing level of kidney function (as assessed with cystatin-C or eGFR) suggesting that β_2_M elevations may have pathologic significance above and beyond its association with glomerular filtration.

These clinical and basic science observations suggest that it is at least possible that β_2_M may be directly, rather than indirectly, e.g. as a surrogate of RRF, implicated in the heightened morbidity and mortality in ESRD. Thus, combination of the population kinetic model with concentration-effect relationships, known as an exposure-clinical response model in clinical pharmacology [65], can be considered a research tool that facilitates quantitative predictions and testable hypotheses to be generated. One such prediction is that total loss of RRF will be associated with worsened survival in HD. For US patients, who initiate Conventional thrice-weekly HD at an average kidney function of 10 ml/min [66] the RR associated with total loss of kidney function is over 20% (>30% for LF dialysis) in our simulations. This is approximately 60% of the corresponding estimate reported in a prospective Dutch cohort [67]. It is tempting to hypothesize that loss of middle molecule clearance is a large component of the heightened mortality risk due to the loss of RRF observed in the real world, while the remaining excess mortality is explained by the imperfect capability of renal replacement therapies to restore fluid, electrolyte and other uremic toxin (e.g. bound solutes) [68–70] homeostasis.

Our simulations predict that higher dialytic removal of β_2_M will not affect the RR for death until RRF has declined below 2ml/min. This was seen for all comparisons based on interventions that have been tested in actual medium-large scale RCTs: HF vs. LF (HEMO [14] and MPO [26]), SD vs. HF [35], LD vs. HF [36], LF vs. HDF (CONTRAST [27]), HF vs. HDF (ESHOL [28] and Turkish trials [29]) as well as trial configurations that to our knowledge have not been reported in the literature: SD or LD vs. HDF. Furthermore, the benefits at the population level that are likely to accrue due to higher middle molecule removal from such interventions are unlikely to be large relative to the de-facto standard of HF dialysis [71] unless treatment time, frequency and possibly convective clearance [72] are all increased. Overall these predictions are consistent with the findings of RCTs in this area, yet suggest that subgroups defined by toxin generation rate may receive benefit more than others. To our knowledge, this hypothesis has never been evaluated in either a RCT or an associational study context. Hence, the availability of a population kinetic model described herein provides an opportunity to directly test this hypothesis by yielding a tool that can be used to characterize kinetics in an individualized manner and use this information in a research setting. This is similar to the use of quantitative models in pharmacology [73] to estimate patient specific variables that are used to individualize plasma drug concentrations or pharmacodynamic responses in research or clinical care settings. In this light, the observation that the predialysis β_2_M concentration is numerically very close to the TAC of β_2_M (at least for dialysis practices that are widely employed in the US), suggests that predialysis β_2_M concentration monitoring may offer some of the benefits of more extensive modeling.

From a practical standpoint, the numerical simulations reported herein support the argument that delaying loss of kidney function should be counted among the therapeutic HD goals. This argument agrees with a large body of emerging clinical data [74] associating RRF with improved survival, lower hospitalizations, improved anemia and phosphorus management, better volume management and decreased left ventricular hypertrophy. Thus, aiming only for “more dialysis” without considering RRF is too narrow of a focus if better outcomes are to be attained. The recent report that frequent, prolonged HD is associated with faster declines in RRF [75] while short frequent sessions [75] or HDF [76] may have a neutral or even positive impact in sustaining kidney function, suggests that a tailored approach to dialysis prescription that considers both dialytic and kidney function is required.

The findings and interpretations in this report should be viewed in light of certain limitations in the source data, and in the kinetic and outcome models used. Firstly, the available kinetic studies of β_2_M involved only a small number of patients and it is possible, that previous research failed to include a representative sample from the human population. Furthermore, our search strategy may have failed to identify all relevant publications. Nevertheless, our simulations reproduced a number of experimental findings so that a strong bias from these two sources is unlikely. Secondly, we have assumed that higher dialytic clearance will affect β_2_M concentrations only through the dialytic removal of the molecule, without affecting its generation rate. Although suggested by some *in vitro* studies [77,78], other ex vivo [79,80] and *in vivo* [81] investigations did not demonstrate an effect of dialyzer flux on β_2_M gene transcription or protein expression, thus providing empiric support for our assumptions regarding this matter. Thirdly, a few studies have raised the possibility that a third compartment [47,82] may be needed to accurately describe β_2_M kinetic behavior in patients undergoing long-term HD (>6 years). Therefore, in this report we limited our simulations to 3 months, an interval much longer than the 2–3 weeks needed to evaluate the effects of a given dialysis procedure [47], but shorter than the time over which the two-pool model would potentially lose its accuracy. Finally, the association between middle molecules and outcomes was assessed using a single biomarker (β_2_M).

One possibility is that β2M, although extensively studied, is an imperfect marker for the removal of the entire spectrum of the peptides/proteins of molecular weight >500 kDa classified as middle molecules. [53,72] Notwithstanding the observations that other middle molecules (beta trace protein [83] or cystatin-C [84]) have also been linked to HD outcomes this is a major limitation due to gaps in the existing literature that only further experimental research can resolve. For example, other uremic solute categories (such as the protein bound toxins) may even be more important than middle molecules and these are more efficiently cleared by larger dialyzers in prolonged sessions [68] or by HDF [69,85]. Supporting recent appraisals and criticisms [86–88], these considerations suggest the need for a more rigorous evaluation of longitudinal changes in a number of candidate uremic toxins in relation to patient outcomes beyond β_2_M. Such a task may be facilitated by targeted analyses in existing biorepositories [89,90] established in the context of RCTs or through establishment of collaborative efforts [91] in prospectively followed observational cohorts. [83]

In summary, we have undertaken a quantitative analysis of the available kinetic studies of β_2_M in order to simulate β_2_M concentrations and associated changes in survival across a wide range of dialysis regimes ranging from conventional thrice weekly HD to long daily sessions with HF dialyzers and HDF. These simulations support many clinical observations over the last 30 years while suggesting that optimal middle molecule dialysis may critically depend on the preservation of RRF. Future studies should examine the validity of these predictions against non-standard schedules of frequent short and long HD and HDF and test the utility of the estimated population model in individualizing treatment parameters.

## Supporting Information

S1 Fig Compartmental kinetic model of β_2_M metabolism. **A)** Bi-compartmental system describing β_2_M kinetics consisting of a plasma/perfusing (P) and non-perfusing/non-plasma (NP) with additional material fluxes for patients during hemodialysis sessions (stippled shapes). In each compartment, the symbols V, Φ, C denote the absolute and fractional volume of each compartment and the concentration of β2M respectively. Generation (G) takes place in both compartments, in direct proportion to their fractional volumes. *K*_*D*_, *K*_*ER*_, *K*_*R*_ are the dialyzer clearance, extrarenal and residual renal clearances. **B)** System differential equations for patients receiving dialysis (variable volume model). Volume changes during dialysis (Θ = 1) as a result of ultrafiltration (*Q*_*UF*_), as well as in the interdialytic intervals (Θ = 0) due to fluid intake (*α*).
(TIFF)

S1 Table Data extraction form and individual subject data extracted from the studies of the systematic review (CSV)

S2 Table Research reports identified through PUBMED search until June 2015. (XLS)

S1 Text Detailed descriptions of the differential equations of the β2M bicompartmental, variable volume model and the numerical aspects of the simulations. (DOCX)

S2 Text PRISMA checklist for the systematic review of studies concerning the kinetics of beta 2 microglobulin. (DOC)
