# Supplementary figures and images for: Revisiting the Middle Molecule Hypothesis of Uremic Toxicity: A Systematic Review of Beta 2 Microglobulin Population Kinetics and Large Scale Modeling of Hemodialysis Trials In Silico
Source: PLoS One. 2016 Apr 7;11(4):e0153157. doi: 10.1371/journal.pone.0153157 (PMC4824495; doi:10.1371/journal.pone.0153157)

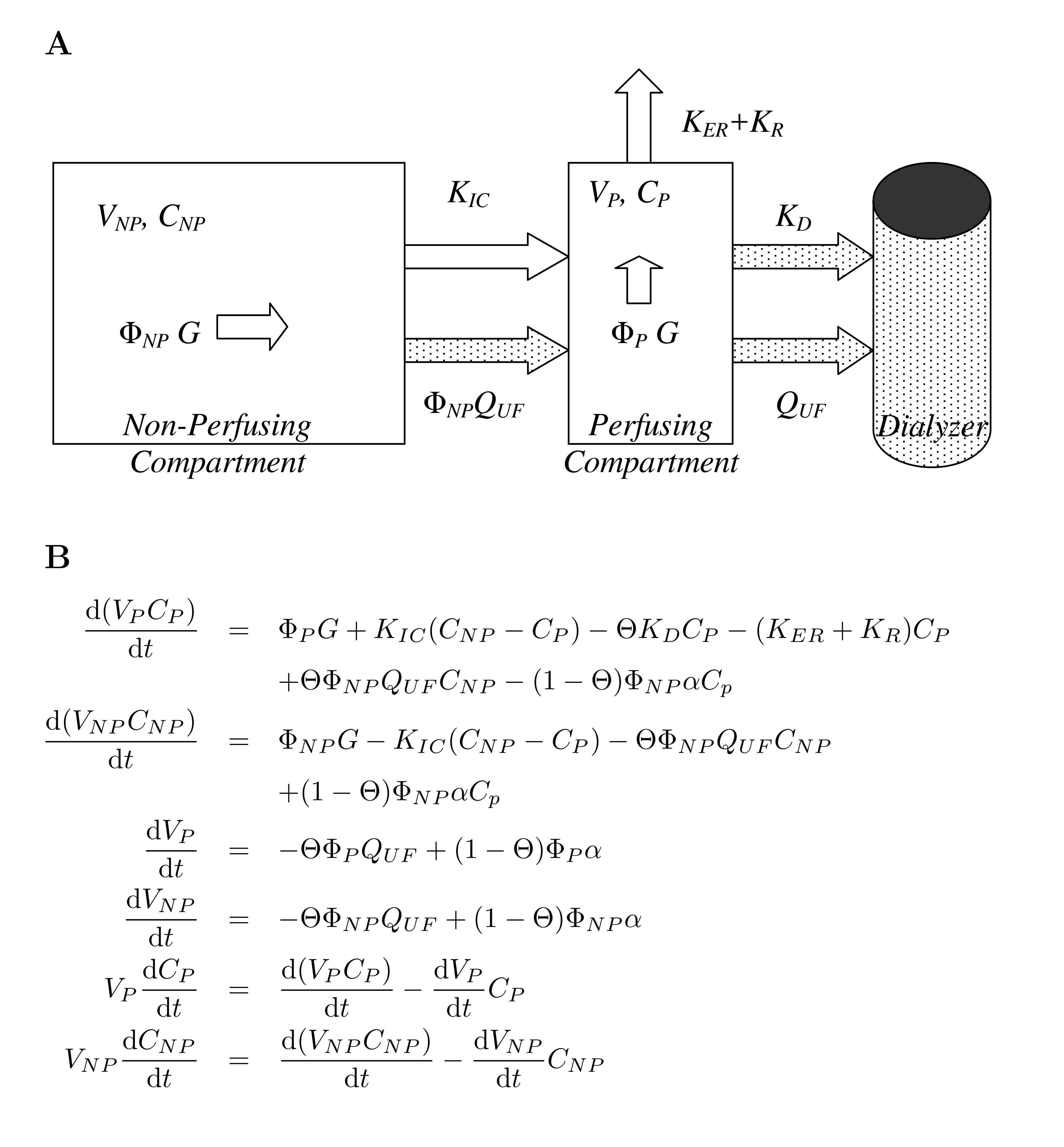

Supplement: S1 Fig — A) Bi-compartmental system describing β2M kinetics consisting of a plasma/perfusing (P) and non-perfusing/non-plasma (NP) with additional material fluxes for patients during hemodialysis sessions (stippled shapes). In each compartment, the symbols V, Φ, C denote the absolute and fractional volume of each compartment and the concentration of β2M respectively. Generation (G) takes place in both compartments, in direct proportion to their fractional volumes. KD, KER, KR are the dialyzer clearance, extrarenal and residual renal clearances. B) System differential equations for patients receiving dialysis (variable volume model). Volume changes during dialysis (Θ = 1) as a result of ultrafiltration (QUF), as well as in the interdialytic intervals (Θ = 0) due to fluid intake (α). (TIFF) [file pone.0153157.s001.tiff]
