# Supplementary material for: Revisiting the Middle Molecule Hypothesis of Uremic Toxicity: A Systematic Review of Beta 2 Microglobulin Population Kinetics and Large Scale Modeling of Hemodialysis Trials In Silico
Source: PLoS One. 2016 Apr 7;11(4):e0153157. doi: 10.1371/journal.pone.0153157 (PMC4824495; doi:10.1371/journal.pone.0153157)
Supplement: S1 Text — (DOCX) [file pone.0153157.s004.docx]

## Supplementary Methods

β2M kinetic simulations were based on variable volume model with two compartments: a *perfusing* (*central, plasma*) and *a non-perfusing* (*peripheral, tissue, extravascular*) one with volumes of distribution *V_P_* and *V_NP_* respectively (S4 Fig.). Generation (*G*) occurs proportionally to the volume of each compartment, while β_2_M distributes between the two pools by both diffusion (intercompartmental transfer constant *K_IC_*) and convection (volume shifts during dialysis and in the interdialytic interval). β_2_M is eliminated from the body by combined renal (*K_R_*), extrarenal (*K_ER_*) and dialytic (K_D_) routes from the perfusing compartment only. K_D_ has both convective components (K_conv_, minimal except in patients receiving postdilution hemodiafiltration) and non-convective components comprising of both diffusive and possibly adsorptive components. Changes in the volumes of the two compartments occur during dialysis (ultrafitration, rate *Q_UF_*) and in the interdialytic period (fluid intake, with rate *α*) so that the relative volume (*V_P_/V_NP_*) stays constant both intra (Θ=1) and inter-dialytically (Θ=0).

*Simulations:* We simulated the kinetic parameters (*G, K_IC_,* Φ*_P_,* Φ*_NP_*, *V_T_*, *K_ER_* ,*K_R_* ) of 10000 patients from the scaled–for–weight distributions of the population mean and standard deviation(sd) estimated from the literature synthesis and different levels of residual renal function (RRF: 0 to 10 ml/min). β_2_M trajectories were simulated over three months under conventional (thrice weekly) dialysis with Low Flux (LF) or High Flux (HF) membranes, short (SD) or long (LD) daily (six times/week) dialysis with HF membranes and thrice weekly online hemodiafiltration (HDF) by solving the differential equations in Supplementary Figure 1 with the LSODA integrator.^1^ To calculate β_2_M TAC (Time Averaged Concentration), the system of differential equations was solved for an entire week under a fixed sampling scheme in order to generate concentrations every 10 minutes. These measurements were subsequently integrated with the standard trapezoidal rule to compute the Area Under the Curve (AUC) which was then divided by the number of minutes in a week in order to yield the TAC.

Dialysis session parameters and anthropometrics (mean ± sd) reflected the patterns observed in North American ESRD participants of randomized trials^2–4^: a) treatment duration: 213 ± 28 mins (LF/HF/HDF), 154 ± 25 min (SD), 379 ± 62 mins (LD) b) interdialytic weight gain: 2.5 ± 0.8 L (LF/HF/HDF), 2.1 ± 0.7 L(SD), 1.9 ± 0.66L (LD) c) dry patient weights 78 ± 20 kgr (all regimes). Dialyzer clearance was based on the largest to date analysis of membrane performance which was undertaken during the HEMO trial^5^: average LF clearance 3.5 ml/min, HF clearance: 35 ± 10 ml/min (restricted to lie in the 20-80 ml/min range). These estimates of plasma water clearance, derived from blood side measurements pre and post dialysis are averages of the adsorptive and diffusive components of dialytic clearance over an entire session. As dialyzer performance declines over the course of a session, average clearance is smaller^6–9^ than instantaneous estimates reported at specific time points during a treatment.^10,11^ Under these assumptions of the distribution of dialytic clearance, the simulated patients were exposed to high flux dialyzers with performance typical of Class II ( “standard high flux” up to 30 ml/min, 25% of patients), Class II (“modern high flux” 30-50 ml/min, 68% of patients) and Class 4 (“super high flux”, 50-70 ml/min 7% of patients) in the Japanese Ministry of Labor and Welfare classification and reimbursement scale for dialysis procedures^12^ For HDF additional determinants of convective removal were based on weighted average values in the Dutch CONTRAST^13^, Spanish ESHOL^14^ and the Turkish OL-HDF^15^ RCTs: treatment duration: 233 ± 4.6 min Qb (blood flow): 358 ± 19 ml/min (giving more weight to ESHOL which had used blood flow rates more typical of North American dialysis practices) , Qinf (infusion volume): 19.2 ± 2.3 L/session under the constraint that the filtration fraction (the ratio of the total convection volume, i.e. the sum of ultrafiltration and infusion volumes, to blood flow rate) lies within 20-30% with the upper half of this range achievable by automatic adjustment of the transmembrane pressure^16^. The latter constraint stems from the need to avoid excessive hemoconcentration and appeared to have been observed in the 3 HDF trials on the basis of the published data of blood flow rate, duration and total convection volumes (26.6%, 26% and 26.3% in CONTRAST, Turkish and ESHOL). The distribution of filtration fractions in the simulations (mean ± sd of 27 ±1.8%) was similar to the one in the 3 HDF trials after accounting for the higher ultrafiltration volumes characteristic of the larger (by approximately 10 kgr) FHN patients relative to the European participants in the HDF studies. Hematocrit (Hct), which influences Kconv, reflected the most recent hemoglobin (Hb) trends in the USRDS^17^, 10.8 ± 1.1 g/dl, converted to Hct by the formula: $Hct=Hb\times3$. Membrane sieving coefficients were simulated assuming a mean/standard deviation of 0.72 ± 0.06 so that the majority of the sieving coefficients used in these simulations were above 0.6 and 0.67 (98% and 80% respectively) typical of membranes used in modern HDF^18^. These parameters were used to derive the convective component using standard formulas^16^ that assume a competition between convective and non-convective removal expressed as a transmittal coefficient^19,20^ that scales the total convection rate of the session. This coefficient^21^ is a function of the operational plasma flow rate, the sieving coefficient and the clearance at zero ultrafiltration rate (K_D_), so that membranes with smaller K_D_ experience the largest transmittal. In our HDF simulations the total clearance exceeded the clearance of the same dialyzer used in HF mode by a factor of 2.56 ± 0.35. This figure is compatible with the range established in previous studies^22–24^ after allowing for differences in measurement procedures (blood side vs dialysate side), convection and blood flow rates employed.

*Supplementary References*

1. Petzold L. Automatic Selection of Methods for Solving Stiff and Nonstiff Systems of Ordinary Differential Equations. *SIAM J. Sci. Stat. Comput.* 1983; **4**: 136–148.

2. Eknoyan G, Beck GJ, Cheung AK, *et al.* Effect of Dialysis Dose and Membrane Flux in Maintenance Hemodialysis. *N Engl J Med* 2002; **347**: 2010–2019.

3. Chertow GM, Levin NW, Beck GJ, *et al.* In-center hemodialysis six times per week versus three times per week. *N. Engl. J. Med.* 2010; **363**: 2287–2300.

4. Rocco MV, Lockridge RS Jr, Beck GJ, *et al.* The effects of frequent nocturnal home hemodialysis: the Frequent Hemodialysis Network Nocturnal Trial. *Kidney Int.* 2011; **80**: 1080–1091.

5. CHEUNG AK, AGODOA LY, DAUGIRDAS JT, *et al.* Effects of Hemodialyzer Reuse on Clearances of Urea and {beta}2-Microglobulin. *J Am Soc Nephrol* 1999; **10**: 117–127.

6. Padrini R, Canova C, Conz P, *et al.* Convective and adsorptive removal of β2-microglobulin during predilutional and postdilutional hemofiltration. *Kidney Int.* 2005; **68**: 2331.

7. Pedrini LA, De Cristofaro V, Pagliari B, *et al.* Mixed predilution and postdilution online hemodiafiltration compared with the traditional infusion modes. *Kidney Int.* 2000; **58**: 2155.

8. Pedrini LA, Cozzi G, Faranna P, *et al.* Transmembrane pressure modulation in high-volume mixed hemodiafiltration to optimize efficiency and minimize protein loss. *Kidney Int.* 2006; **69**: 573–579.

9. Ouseph R, Hutchison CA, Ward RA. Differences in solute removal by two high-flux membranes of nominally similar synthetic polymers. *Nephrol Dial Transpl.* 2008; **23**: 1704–1712.

10. Krieter DH, Hackl A, Rodriguez A, *et al.* Protein-bound uraemic toxin removal in haemodialysis and post-dilution haemodiafiltration. *Nephrol. Dial. Transplant.* 2010; **25**: 212–218.

11. Bhimani JP, Ouseph R, Ward RA. Effect of increasing dialysate flow rate on diffusive mass transfer of urea, phosphate and β2-microglobulin during clinical haemodialysis. *Nephrol. Dial. Transplant.* 2010.

12. Yamashita AC. Mass Transfer Mechanisms in High-Performance Membrane Dialyzers. In: Saito A, Kawanishi H, Yamashita AC, *et al.*, eds. *Contributions to Nephrology*.Vol 173. Basel: KARGER; 2011:95–102.

13. Grooteman MPC, van den Dorpel MA, Bots ML, *et al.* Effect of online hemodiafiltration on all-cause mortality and cardiovascular outcomes. *J. Am. Soc. Nephrol. JASN* 2012; **23**: 1087–1096.

14. Maduell F, Moreso F, Pons M, *et al.* High-efficiency postdilution online hemodiafiltration reduces all-cause mortality in hemodialysis patients. *J Am Soc Nephrol* 2013; **24**: 487–97.

15. Ok E, Asci G, Toz H, *et al.* Mortality and cardiovascular events in online haemodiafiltration (OL-HDF) compared with high-flux dialysis: results from the Turkish OL-HDF Study. *Nephrol Dial Transpl.* 2013; **28**: 192–202.

16. Tattersall JE, Ward RA, EUDIAL group. Online haemodiafiltration: definition, dose quantification and safety revisited. *Nephrol. Dial. Transplant. Off. Publ. Eur. Dial. Transpl. Assoc. - Eur. Ren. Assoc.* 2013; **28**: 542–550.

17. System USRD. USRDS 2012 Annual Data Report: Atlas of Chronic Kidney Disease and End-Stage Renal Disease in the United States,. 2012.

18. Hoenich NA. Membranes and Filters for Haemodiafiltration. In: Ronco C, Canaud B, Aljama P, eds. *Contributions to Nephrology*. Basel: KARGER; 2007:57–67.

19. Waniewski J. Mathematical modeling of fluid and solute transport in hemodialysis and peritoneal dialysis. *J. Membr. Sci.* 2006; **274**: 24–37.

20. Leypoldt JK. Solute fluxes in different treatment modalities. *Nephrol. Dial. Transplant. Off. Publ. Eur. Dial. Transpl. Assoc. - Eur. Ren. Assoc.* 2000; **15 Suppl 1**: 3–9.

21. Ficheux A, Argilés À, Mion H, *et al.* Influence of convection on small molecule clearances in online hemodiafiltration Technical Note. *Kidney Int.* 2000; **57**: 1755.

22. Lornoy W, Becaus I, Billiouw JM, *et al.* Remarkable removal of beta-2-microglobulin by on-line hemodiafiltration. *Am. J. Nephrol.* 1998; **18**: 105–108.

23. Ward RA, Schmidt B, Hullin J, *et al.* A Comparison of On-Line Hemodiafiltration and High-Flux Hemodialysis: A Prospective Clinical Study. *J. Am. Soc. Nephrol.* 2000; **11**: 2344–2350.

24. Anchana Panich KT. The effectiveness of on-line hemodiafiltration on beta-2 microglobulin clearance in end stage renal disease. *J. Med. Assoc. Thail. Chotmaihet Thangphaet* 2006; **89 Suppl 2**: S1–8.
