# Supplementary material for: Revisiting the Middle Molecule Hypothesis of Uremic Toxicity: A Systematic Review of Beta 2 Microglobulin Population Kinetics and Large Scale Modeling of Hemodialysis Trials In Silico
Source: PLoS One. 2016 Apr 7;11(4):e0153157. doi: 10.1371/journal.pone.0153157 (PMC4824495; doi:10.1371/journal.pone.0153157)
Supplement: S2 Text — (DOC) [file pone.0153157.s005.doc]

| **Section/topic** | **#** | **Checklist item** | **Reported on page #** |
| --- | --- | --- | --- |
| **TITLE** | | |  |
| Title | 1 | The study title clearly identifies this as a systematic review | Page 1 |
| **ABSTRACT** | | |  |
| Structured summary | 2 | Provide a structured summary including, as applicable: background; objectives; data sources; study eligibility criteria, participants, and interventions; study appraisal and synthesis methods; results; limitations; conclusions and implications of key findings; systematic review registration number. |  |
| **INTRODUCTION** | | |  |
| Rationale | 3 | Second paragraph of introduction | Page 3-4 |
| Objectives | 4 | This report aims to develop a population kinetic model incorporating the intra-individual variability in generation, distribution and extrarenal elimination of β2M, which is then used to describe the disposition of β2M under different HD regimes and levels of RRF. To develop this population kinetic model, we first undertook a patient-level review and synthesis of the literature of clinical studies (either observational or interventional) regarding these kinetic parameters of β2M in humans. | Page 4 |
| **METHODS** | | |  |
| Protocol and registration | 5 | As this was not an systematic review for a specific health outcome, we did not register our study in a prospective fashion. | Page 4 |
| Eligibility criteria | 6 | We included studies published in Medline (up to June 2010) if they had used a compartmental model to study β2M kinetics, reported patient-level data and were published in English prior to June 2010.  We did not look for studies past 2010, as a number of important randomized trials examining novel forms of delivering dialysis were scheduled to be finished and published after that date. The outcomes of these studies would then validate our predictions in a *prospective fashion* | Page 4 |
| Information sources | 7 | Medline (up to June 2010) | Page 4 |
| Search | 8 | The text sting used to search Medline is provided in the text | Page 4 |
| Study selection | 9 | We examined the full text of all studies indentified through the Medline search, to exclude those that were in-vitro investigations, simulation experiments, failure to employ a kinetic model, or reported aggregate rather than subject level data (exclusion criteria). | Page 4 |
| Data collection process | 10 | A piloted form was used to extract the individual data from each study jointly by two investigators. This form and the corresponding database were uploaded as S2 Table. | Page 5 |
| Data items | 11 | Values of kinetic parameters about the generation, intra-compartmental distribution, volume of compartments, non-renal clearance of β2M. Additional methods were given in S1 Text | Page 5 |
| Risk of bias in individual studies | 12 | To our knowledge there is no available method for the analysis of bias in studies that lack a discrete health outcome. Hence we could not undertake a formal analysis of bias e.g. with a funnel plot, as these are not defined for the types of studies we considered. However for each study we evaluated the number of parameters reported, those fixed and those unreported by the investigators as an indicator for bias. We considered studies that did not report (or fixed to a specific value) of at most one parameter as studies with minimal risk of bias. As the number of parameters with fixed (or unreported values) increases, the estimated values of the remaining parameters becomes more and more dependent on the specific assumptions of the investigators and thus the risk for bias increases. | Page 5 |
| Summary measures | 13 | Results are reported as means (SE) for the mean and the logarithm of the standard deviation of each log-transformed kinetic parameter. | Page 6 |
| Synthesis of results | 14 | We analyzed studies that collected multiple measurements in the same individual with a bi-level mixed-effect model accounting for individual (first level) and study (second level) heterogeneity; all other studies were analyzed with a random-effects model with a single (study) random effects model. Parameters were log-transformed prior to mixed-effect modeling of the population mean and (log-) variance, which was estimated by the between individual (two level models) or within study (one level model) standard error. | Page 6 |

Page 1 of 2

| **Section/topic** | **#** | **Checklist item** | **Reported on page #** |
| --- | --- | --- | --- |
| Risk of bias across studies | 15 | We considered studies that did not report (or fixed to a specific value) of at most one parameter as studies with minimal risk of bias. As the number of parameters with fixed (or unreported values) increases, the estimated values of the remaining parameters becomes more and more dependent on the specific assumptions of the investigators and thus the risk for bias increases. | Page 5 |
| Additional analyses | 16 | No further analyses were done | Page 6 |
| **RESULTS** | | |  |
| Study selection | 17 | A flow diagram was provided as Fig 1. We also uploaded the individual studies | Page 7 |
| Study characteristics | 18 | See Table 1 | Page 8 |
| Risk of bias within studies | 19 | Four out of the nine studies were not at risk of bias | Page 7 |
| Results of individual studies | 20 | Not applicable – data from each participant from every study were uploaded in the journal website as S2 Text |  |
| Synthesis of results | 21 | Table 2. | Page 15 |
| Risk of bias across studies | 22 | Four out of the nine studies were not at risk of bias | Page 7 |
| Additional analysis | 23 | Not applicable since none were undertaken |  |
| **DISCUSSION** | | |  |
| Summary of evidence | 24 | Not applicable as this systematic review did not concern specific health-care interventions and their effect on patient outcomes |  |
| Limitations | 25 | Complicated Research string that may have resulted in an incomplete identification of available studies. Small number of studies and individuals in the quantitative evidence synthesis | Pages 27,28 |
| Conclusions | 26 | In summary, we have undertaken a quantitative analysis of the available kinetic studies of β2M in order to simulate β2M concentrations and associated changes in survival across a wide range of dialysis regimes ranging from conventional thrice weekly HD to long daily sessions with HF dialyzers and HDF. These simulations support many clinical observations over the last 30 years while suggesting that avoidance of middle molecule toxicity may critically depend on both the preservation of RRF and tailoring of therapy to patient profiles (e.g. intrinsic generation rate). Furthermore these observations underscore practical limitations (study size, duration of follow up) in validating the middle molecule hypothesis in unselected patients with ESRD. Future studies should examine the validity of these predictions against non-standard schedules of frequent short and long HD and HDF and test the utility of the estimated population model in individualizing treatment parameters. | Page 28 |
| **FUNDING** | | |  |
| Funding | 27 | As stated in the electronic submission, no funding was provided for this study |  |

*From:*  Moher D, Liberati A, Tetzlaff J, Altman DG, The PRISMA Group (2009). Preferred Reporting Items for Systematic Reviews and Meta-Analyses: The PRISMA Statement. PLoS Med 6(6): e1000097. doi:10.1371/journal.pmed1000097

For more information, visit: **www.prisma-statement.org**.

Page 2 of 2
